# Editorial: New Approaches to Tackle EMT and Fibrosis: From Epigenetics to Nanotechnology

**DOI:** 10.3389/fphar.2021.742777

**Published:** 2021-08-06

**Authors:** Cecilia Battistelli, Marco Cordani, Marc Diederich, Álvaro Somoza, Sergio Valente, Raffaele Strippoli

**Affiliations:** ^1^ Department of Molecular Medicine, Sapienza University of Rome, Rome, Italy; ^2^ IMDEA Nanociencia, Madrid, Spain; ^3^ Department of Pharmacy, College of Pharmacy, Seoul National University, Seoul, South Korea; ^4^ Department of Chemistry and Technologies of Drugs, Sapienza University of Rome, Rome, Italy; ^5^ Istituto Nazionale per le Malattie Infettive Lazzaro Spallanzani (IRCCS), Rome, Italy

**Keywords:** EMT—epithelial to mesenchymal transition, epigenetics, nanotechnology, fibrosis, cancer biology

Epithelial-mesenchymal transition (EMT) is a highly dynamic, multistep process implicated in various physio-pathological conditions, including chronic inflammation, fibrogenic diseases, and cancer. In tumors, the acquisition of EMT provides increased migratory-invasive abilities. More recently, EMT was shown to mediate the acquisition of chemoresistance linked to cancer stem cell (CSC)-like features.

Due to its role in the pathogenesis of fibrotic diseases and cancer, growing evidence suggests that the pharmacological inhibition of EMT may be a valid therapeutic approach. However, attempts to target EMT per se or in combination with other treatments failed in most cases. One possible explanation may reside in incomplete knowledge of the underlying mechanisms or the lack of appropriate systems for delivering EMT-targeting therapies. Indeed, the standard therapeutic strategies may present various drawbacks, such as low specificity, drug resistance, rapid drug clearance, and biodegradation, leading to treatment failure.

The scope of this research topic is to provide an updated overview on nano-therapeutic strategies focused on epigenetics and aimed to target EMT processes.

Tanabe et al. analyzed the interplay between EMT and CSC populations, emphasizing EMT regulation by microRNAs. CSCs consist of cancer cells with stem-like features, which have capacities of self-renewal and differentiation in cancer cells. Some populations of CSCs share EMT-like cell features, which is linked to increased chemoresistance. The rationale of linking CSC to innovating, nanotechnological therapeutic approaches is the resistance of CSCs to conventional chemo- and radiotherapies. Tanabe et al. discussed nanomedical formulations applied to CSC therapy, including polymeric micelle-based nanomedicines incorporating cisplatin (CDDP/m), the combination of thermo- and chemotherapy utilizing multifunctional magnetic nanoparticles, or nanomedicine-containing miRNAs.

A review article by Dong et al. focused on the elucidation of epigenetic mechanisms that modulate EMT, emphasizing the use of epidrugs. Pharmacological inhibitors targeting DNA methyltransferases (DNMTs), histone deacetylases (HDAC), acetylation readers such as bromodomain-containing proteins (BRDs), lysine methyltransferases (KMTs), and histone-specific demethylase 1 (LSD1) were discussed. Moreover, examples of approaches that target long non-coding RNAs (LncRNAs) with RNA interference, antisense oligonucleotides (ASO)-based therapies, lncRNA mimics, and small molecule inhibitors were reported.

Besides cancer, EMT may be targeted pharmacologically in non-tumoral fibrotic diseases, including lung and kidney fibrosis. Xie and Zeng dealt with the therapeutic potential of exosomes on pulmonary fibrosis. Exosomes and their cargos, including miRNAs, lncRNAs, and proteins, can promote or inhibit EMT and modulate fibroblasts’ transformation into myofibroblasts. Indeed, exosomes are key factors regulating the differentiation of bone marrow mesenchymal stem cells (BMSCs) into myofibroblasts. Interestingly, exosomes from BMSCs may improve alveolar epithelial wound repair, inhibit alveolar EMT, and significantly inhibit silicosis. Mechanistically, BMCs may promote the proliferation of type II alveolar epithelial cells, inhibit type II alveolar epithelial fibrosis, and inhibit collagen deposition.

Kim et al. developed a novel therapeutic approach with protein transduction domain (PTD) fused bone morphogenetic protein-7 (BMP-7) in micelle (mPTD-BMP-7) for long-range signaling *in vivo*. As a member of the TGF-β superfamily, BMP-7 plays an essential role as an endogenous antagonist of TGF-β, inhibiting fibrotic progression in many organs. The authors also performed an intra-arterial administration of mPTD-BMP-7 through the renal artery in pigs. Of note, mPTD-BMP-7 was effectively delivered into the Bowman’s space and inhibited unilateral ureter obstruction–induced renal fibrosis.

Skibba et al. provided a comprehensive review of nano-approaches to target epigenetic mechanisms for the treatment of pulmonary fibrosis. The authors first elucidated the primary cellular and molecular mechanism mediating EMT and myofibroblast transition in lung fibrosis.

Moreover, they analyzed epigenetic mechanisms focusing on the role of bromodomain-containing protein 4 (BRD4). Then, the main therapeutical strategies for treating pulmonary diseases, including liposomes, polymeric nanoparticles (NPs), inorganic NPs, and exosomes, were discussed.

Last, Shen et al. analyzed the effect of sodium hottuyfonate (SH), an addition compound from *Houttuynia cordata* Thunb., in bleomycin-induced pulmonary fibrosis in mice. The authors found that SH attenuated BLM-induced lung injury. Moreover, they linked these *in vivo* effects with the inhibition of TGF-β1 signaling and to a reduced secretion of pro-inflammatory cytokines.

In conclusion, manuscripts included in this special number show that the use of drug delivery systems based on nanomaterials improved the properties of various bioactive therapies, including drugs, peptides, and antibodies. Also, advances in material sciences shown here will allow the development of different nanomaterials to improve the biodistribution of drugs and their accumulation at the target site. So far, both preclinical and clinical studies presented here have provided encouraging results.

